# Investigating the Effect of Cochlear Synaptopathy on Envelope Following Responses Using a Model of the Auditory Nerve

**DOI:** 10.1007/s10162-019-00721-7

**Published:** 2019-05-17

**Authors:** Gerard Encina-Llamas, James M. Harte, Torsten Dau, Barbara Shinn-Cunningham, Bastian Epp

**Affiliations:** 1 Hearing Systems section, Department of Health Technology, Technical University of Denmark (DTU), Kongens Lyngby, Denmark; 2 Interacoustics Research Unit, Kongens Lyngby, Denmark; 3 Carnegie Mellon Neuroscience Institute, Pittsburgh, PA USA; 4 Department of Biomedical Engineering, Boston University, Boston, MA USA

**Keywords:** cochlear synaptopathy, “hidden” hearing loss, envelope following responses, auditory steady-state responses, auditory nerve modeling

## Abstract

The healthy auditory system enables communication in challenging situations with high levels of background noise. Yet, despite normal sensitivity to pure tones, many listeners complain about having difficulties in such situations. Recent animal studies demonstrated that noise overexposure that produces temporary threshold shifts can cause the loss of auditory nerve (AN) fiber synapses (i.e., cochlear synaptopathy, CS), which appears to predominantly affect medium- and low-spontaneous rate (SR) fibers. In the present study, envelope following response (EFR) magnitude-level functions were recorded in normal hearing (NH) threshold and mildly hearing-impaired (HI) listeners with thresholds elevated above 2 kHz. EFRs were elicited by sinusoidally amplitude modulated (SAM) tones presented in quiet with a carrier frequency of 2 kHz, modulated at 93 Hz, and modulation depths of 0.85 (deep) and 0.25 (shallow). While EFR magnitude-level functions for deeply modulated tones were similar for all listeners, EFR magnitudes for shallowly modulated tones were reduced at medium stimulation levels in some NH threshold listeners and saturated in all HI listeners for the whole level range. A phenomenological model of the AN was used to investigate the extent to which hair-cell dysfunction and/or CS could explain the trends observed in the EFR data. Hair-cell dysfunction alone, including postulated elevated hearing thresholds at extended high frequencies (EHF) beyond 8 kHz, could not account for the recorded EFR data. Postulated CS led to simulations generally consistent with the recorded data, but a loss of all types of AN fibers was required within the model framework. The effects of off-frequency contributions (i.e., away from the characteristic place of the stimulus) and the differential loss of different AN fiber types on EFR magnitude-level functions were analyzed. When using SAM tones in quiet as the stimulus, model simulations suggested that (1) EFRs are dominated by the activity of high-SR fibers at all stimulus intensities, and (2) EFRs at medium-to-high stimulus levels are dominated by off-frequency contributions.

**Electronic supplementary material:**

The online version of this article (10.1007/s10162-019-00721-7) contains supplementary material, which is available to authorized users.

## Introduction

It is well known that noise overexposure can impair the auditory system by producing a sensorineural hearing loss, seen in a permanent elevation of pure-tone detection thresholds. This has led to the interpretation that sound stimulation producing only a temporary threshold shift (TTS), but not a permanent threshold shift (PTS), does not permanently damage the auditory system. However, it has been reported that, despite normal sensitivity to pure tones, some listeners complain about having listening difficulties in challenging acoustical situations (Hind et al. 2011; Kumar et al. 2007; Saunders and Haggard 1989; Tremblay et al. 2015).

Recent animal studies have shown that noise overexposure producing TTS can in fact lead to the loss of AN fiber synapses, without damaging the sensitive hair cells in the cochlea Kujawa and Liberman (2009). As this neuronal degeneration does not result in a PTS, it has been termed “hidden” hearing loss (Schaette and McAlpine 2011). (Kujawa and Liberman 2009) demonstrated in mice that “hidden” hearing loss, or more accurately cochlear synaptopathy (CS) (for a review, see Liberman and Kujawa 2017), resulting from carefully controlled noise exposure, did not alter hearing thresholds. It was further shown that the magnitude-level function of distortion-product otoacoustic emissions (DPOAE) remained unaffected in the same mice. These results indicate that the outer hair cells (OHC) were not damaged due to the noise exposure. The amplitude of the auditory brainstem response (ABR) wave-I, on the other hand, was reduced at supra-threshold sound pressure levels (SPL). Wave-I is thought to reflect the action potentials of the AN, and should therefore be sensitive to a loss of AN fiber synapses. It has been suggested that a *selective* (meaning *predominant*) loss of medium- and low-spontaneous rate (SR) fibers could account for the reduction of supra-threshold ABR wave-I magnitudes, while still preserving normal thresholds (Furman et al. 2013). A reanalysis of the data from Furman et al. (2013) concluded that there was indeed a loss of high-SR fibers at a ratio of about 1:3 with loss of low- and medium-SR fibers (Marmel et al. 2015). Thus, although medium- and low-SR fibers may be more affected than high-SR fibers, all fibers are likely affected to some degree. In fact, Bourien et al. (2014) showed that changes in ABR wave-I amplitudes are more likely to be due to loss of high-SR fibers than of medium- and low-SR fibers. Additionally, Lobarinas et al. (2013) reported that, even in the case of a substantial loss of inner hair cells (IHC) and AN fibers, behavioral pure-tone thresholds remained unchanged, suggesting that even a substantial loss of high-SR fibers would not produce PTS. Nevertheless, many hypotheses about CS in humans (including this study) start with an assumption that low-SR fibers are more affected than other fibers and that the spiking rate of the high-SR fibers saturates at supra-threshold levels (e.g., Bharadwaj et al. 2015; Mehraei et al. 2016; Paul et al. 2017; Valero et al. 2018).

Noise-induced CS has been observed in several non-human mammalian species, such as mice (Furman et al. 2013; Kujawa and Liberman 2009), guinea pigs (Lin et al. 2011; Liu et al. 2012), rats (Lobarinas et al. 2017), and rhesus macaques (Valero et al. 2017). CS has also been reported as a natural phenomenon in the normally aging (non-exposed) mouse ear (Sergeyenko et al. 2013). Noise exposure seems to accelerate this natural degeneration of the AN (Fernandez et al. 2015). In humans, there is some evidence of such age-related CS (Makary et al. 2011; Viana et al. 2015; Wu et al. 2018). Elderly subjects show losses of over 60 % of their synapses compared to younger (Wu et al. 2018). In addition, the loss of peripheral axons in normal aging humans is significantly greater than the loss of spiral ganglion cells (SGC) (Viana et al. 2015), like as reported in mice (Sergeyenko et al. 2013). This suggests that SGC survive for months after the loss of their peripheral axons (Kujawa and Liberman 2015). However, clear evidence of noise-induced CS in living humans has not yet been proven, and the potential perceptual consequences remain unknown (Oxenham 2016; Plack et al. 2014), despite attempts to identify them in large studies (e.g., Grose et al. 2017; Le Prell et al. 2018; Lopez-Poveda et al. 2017; Prendergast et al. 2017).

Animal studies suggest that CS is reflected in electroencephalographic (EEG) evoked response measurements, such as ABR wave-I (Furman et al. 2013; Kujawa and Liberman 2009) or envelope following responses (EFR) (Parthasarathy and Kujawa 2018; Shaheen et al. 2015). Some researchers have attempted to relate changes in evoked responses to self-reported estimates of noise exposure in humans (Prendergast et al. 2017). To date, no correlation has been found. However, noise exposure scores derived from self-reported questionnaires of lifetime noise exposure rely on the subjective recall of noisy events. Furthermore, they are generally based on numerous assumptions limiting their reliability (Coughlin 1990). Other studies have found correlations between evoked responses and behavioral measures of temporal processing at supra-threshold levels in individual NH threshold listeners (Bharadwaj et al. 2015; Mehraei et al. 2016). In these studies, poorly performing listeners were hypothesized to suffer from CS. The inconclusive outcome of the human studies can be attributed, in part, to the impossibility of directly assessing the status of the AN fiber synapses in living humans. Non-invasive evoked responses can be performed both in humans and non-human animals. Comparing these measures across different species could help to connect careful experimentally induced CS in non-human animals to its (potential) presence in humans. However, evoked responses measured using surface (scalp) electrodes represent the far-field sum of the activity of large populations of neurons, which might not be sensitive to specific local neuronal damage, or may require carefully designed stimuli and recording techniques to reveal such loss.

In the present study, EFRs were measured as a function of stimulus level using both deep and shallow modulations of SAM tones. The listeners had either normal audiometric thresholds or a mild hearing impairment above 3 kHz. We hypothesized that a preferential loss of medium- and low-SR fibers would reduce the EFR magnitudes at high supra-threshold stimulus levels, whereas the responses at lower levels would remain unaffected. We therefore predicted that depending on whether or not medium- and low-SR fibers were present, the slope of the EFR magnitude-level functions at supra-threshold input levels would differ. We expected that such a reduction or slope change would be more pronounced in the EFR responses elicited by shallowly modulated tones than deeply modulated tones. This was based on the argument that high-intensity shallowly modulated stimuli are preferentially encoded by medium- and low-SR fibers (Bharadwaj et al. 2014; Bharadwaj et al. 2015). For HI listeners, the EFR magnitude-level functions at both modulation depths were recorded with the stimulus presented only at a frequency where listener’s audiograms were within the normal range, to increase the likelihood of the presence of CS. It has been proposed that CS might be a precursor of subsequent hair-cell damage (Kujawa and Liberman 2015; Liberman and Kujawa 2017; Sergeyenko et al. 2013). It was assumed that listeners who already show a threshold elevation (and therefore hair-cell dysfunction) at higher audiometric frequencies potentially suffer from CS at lower audiometric frequencies with normal thresholds.

As the history of noise exposure in both NH threshold listeners and HI listeners in this study is unknown, and given that estimates of lifetime noise exposure have failed to predict CS in humans in previous studies (e.g., Prendergast et al. 2017), the present study focused on individual differences in EFR magnitude-level functions and their potential relation to CS. In order to assist with the interpretation and the potential effect of CS on the obtained EFRs, a computational model of the AN was used to study the effects of a differential loss of the different AN fiber types on the EFR magnitude-level functions. The aim of the study was thus to investigate whether a computational model of the AN with simulated CS can account for individual patterns observed in the EFR magnitude-level functions recorded in audiometrically homogeneous listeners at the stimulus frequencies at which they were excited (below 3 kHz).

## Methods

### Listeners

Thirteen adult listeners (4 females, 36.6 ± 17.0 years) participated in this study, separated into groups of 9 NH (3 females, 26 ± 2.4 years) and 4 HI (1 female, 60.5 ± 6.7 years) listeners. All NH threshold listeners had audiometric thresholds below 15-dB hearing level (HL) at octave frequencies between 125 and 8000 Hz. All HI listeners were selected to have normal hearing (threshold ≤ 20-dB HL) below 3000 Hz and a mild hearing loss at 4000 Hz and above, with audiometric thresholds between 20- and 45-dB HL.

### Apparatus

EFR recordings were performed in a dark, double-walled soundproof, and electrically shielded booth, in which listeners laid on a comfortable clinical bed. The listeners watched a silent movie and were instructed to relax and avoid unnecessary movement. The recording and data analysis routines were implemented in MATLAB (The MathWorks, Inc., Natick, Massachusetts, USA). All acoustic stimuli were generated in MATLAB and presented using PLAYREC 2.1 (Humphrey, R., www.playrec.co.uk, 2008–2014) via a RME Fireface UCX soundcard (sampling rate *f*_s ∣ sound_ = 48 kHz, 24 bits). The stimuli were presented through ER-3A insert earphones (Etymotic Research Inc.), with the contralateral ear blocked with a foam earplug.

EFRs were recorded using a Biosemi ActiveTwo system (sampling rate *f*_s ∣ EFR_ = 4096 Hz, 24 bits). Sixty-four active pin-type electrodes were used following the 10–20 system (American Clinical Neurophysiology Society 2006). The results shown in this study represent the Cz-P10 potential in response to right-ear stimulation, and the Cz-P9 potential in response to left-ear stimulation (similar to vertex to ipsi- and to contra-mastoid montage respectively). These electrode pairs were used rather than other electrode pairs or multi-electrode configurations with complex post-processing (Bharadwaj et al. 2014) because a comparable or better signal quality was found in pilot recordings. Common mode sense (CMS) and driven-right-leg (DRL) electrodes (Metting van Rijn et al. 1990) were placed at the center of the parieto-occipital coronal line (on either side of electrode POz). Conductive electrode gel was applied and the absolute offset voltage was stabilized at < 20 mV for each electrode. The recorded EEG signals were downsampled by a factor of 2, resulting in a final sampling frequency of *f*_s ∣ EFR_ = 2048 Hz. The EEG data were stored to hard disk for offline analysis.

All experiments were approved by the Science-Ethics Committee for the Capital Region of Denmark (reference H-16036391).

### EFR Recordings and Analysis

EFR data were recorded in a single session, which lasted approximately 2 h. EFR magnitude-level functions were recorded in NH threshold listeners using input levels in the range from 34- to 87-dB SPL, presented in a random order, chosen separately for each listener. In all NH threshold listeners, the right ear was stimulated. In the HI group, the ear which better fits the selection criteria was chosen as recording ear.

A single SAM tone was used as the stimulus, with $\mathrm{SAM}(t)=A\cdotp \sin \left(2\pi {f}_ct\right)\cdotp \left(\frac{1+m\cdotp \sin\ \left(2\pi {f}_mt\right)}{2}\right)$, where *A*, *f*_*c*_, *f*_*m*_, *m* ∈ [0, 1] and *t* represents the amplitude, the carrier frequency, the modulation frequency, the modulation index, and time, respectively. The SAM tone had a carrier frequency (*f*_*c*_) of 2005 Hz (referred to as 2000 Hz for simplicity, henceforth) and a modulation frequency (*f*_*m*_) of 93 Hz. Two modulation depths (*m*) were used: “deep” (*m* = 0.85) and “shallow” (*m* = 0.25). The stimuli were calibrated using a B&K 4157 ear simulator to the desired root mean squared (RMS) level. The stimuli were digitally generated as 1-s long epochs and continuously presented to the listener in a loop, where a trigger signal marked the beginning of a new epoch for later averaging. The first trigger indicating the beginning of a recording was sent 10-s after the sound was already presented to the listener to ensure a steady-state neuronal response (Pérez-González and Malmierca 2014; Sumner and Palmer 2012). The total stimulus duration varied with the stimulus intensity in order to achieve a statistically significant EFR signal-to-noise ratio (SNR), based on pilot recordings. Table 1 shows the stimulus duration used for each input level in the EFR recordings. The recorded EEG data were filtered using a fourth-order Butterworth digital band-pass filter with cutoff frequencies of 60 and 400 Hz, applied serially in forward and backward direction to yield zero phase. All recorded epochs with a maximum absolute amplitude that exceeded a voltage threshold of 80 μV in any of the channels were rejected to remove artifacts and noisy events from the average pool. Sixteen 1-s long epochs of EEG data (from the remaining epochs after rejecting the noisy ones) were concatenated to form a trial to achieve a higher frequency resolution in the EFR spectrum analysis. In order to increase the SNR, the 16-s long trials were ensemble weighted averaged as described in (John et al. 2001), where the inverse of the variance on each 1-s long epochs was used as the weight.

**Table 1:** Duration of EFR stimuli for each used input level

Input level (dB SPL)	34	40	46	54	60	66	71	77	81	87
Duration (min)	10.0	8.5	8.5	7.0	7.0	6.5	5.5	5.5	5.5	5.5

A *F* test was used to identify statistically significant responses by comparing the spectral power at the modulation frequency (EFR frequency) to the noise power in the range of 3 Hz below and above the modulation frequency (Dobie and Wilson 1996; Picton et al. 2003). The power ratio (*F-ratio*) was calculated as the power in the EFR frequency bin divided by the averaged power in 3 Hz below and above the modulation frequency (96 bins). The probability (*P*) of the EFR power being different from the noise power can be calculated as *1-F*, with *F* representing the cumulative distribution function of the power ratio. The *F* test was defined to be positive if *p* ≤ 0.01 (F critical value ≤ 4.8333 based on 2, 192 degrees of freedom, SNR > 5.84 dB), implying that the EFR frequency was statistically significantly different from the noise estimate. The *F* test was custom implemented in MATLAB.

The statistical analysis was performed in R 3.2.2 (R Core Team 2015) using a linear mixed-effects model. The model was implemented using the “lme4” R-package, v1.1.18.1 (Bates et al. 2015) and fitted using the “lmerTest” R-package, v3.0.1 following the approach of backward reduction based on the step-wise elimination of non-significant model terms with high *P* values (Kuznetsova et al. 2014; Kuznetsova et al. 2017). The resulting model had three fixed effects variables: the level of stimulation as a continuous independent variable, and the modulation depth and the hearing status as categorical independent variables. Listeners and the interaction between listener and modulation depth were treated as random effects. *F* tests using the Satterthwaites method to approximate the denominator degrees of freedom were used to calculate the *P* values for the fixed effects. The *P* values for the random effects were calculated based on likelihood ratio tests (Kuznetsova et al. 2017). The post hoc analysis was performed through a multiple pairwise contrasts comparison of the estimated-marginal means using the “emmeans” R-package, v1.2.4 (Lenth 2016). The *P* values were adjusted for multiple comparisons using the Tukey method.

### AN Model

A humanized phenomenological AN model, implemented in MATLAB, was used to simulate the activity of the AN (Zilany et al. 2009, 2014). The model fibers were tuned to 200 characteristic frequencies (CF) ranging from 0.2 to 20 kHz, corresponding to equally spaced positions in the basilar membrane (BM) according to the cochlear frequency map for humans (Greenwood 1990). A non-uniform distribution of AN fibers per CF (or IHC) was implemented according to the distribution reported in Spoendlin and Schrott (1989), with the total number of AN fibers set to 32,000, chosen to match the healthy auditory system. About 160 AN fibers synapses were independently simulated at each CF. In the framework of the model, CS was simulated by computing a reduced number of AN fiber synapses at each CF. Frequency-specific synaptic loss was implemented by fixing a given percentage of loss of fibers at single CFs, which were interpolated using a shape-preserving piecewise cubic Hermite interpolating polynomial evaluated over the complete range of modeled CFs. Hair-cell impairment was implemented by fitting the listener’s audiogram using the *fitaudiogram2* MATLAB function implemented by Zilany et al. (2009). As the distribution of the different AN fiber types at each CF is unknown in humans, the distribution reported from cats was used: 61 % of high-SR fibers, 23 % of medium-SR fibers, and 16 % of low-SR fibers (Liberman 1978). Model simulations were performed using the same stimuli as in the human EFR recordings but with a duration of 1.2-s to reduce lengthy computational time. Stimulus levels ranged from 10- to 100-dB SPL, in steps of 5 dB.

The model allows for control of the IHC and OHC function independently, and provides the deterministic IHC voltage and the stochastic synaptic output of each AN fiber type separately. The same IHC voltage at each CF was used to drive the synapse- and spike generator models (see Zilany et al. 2009), which was executed independently once for each AN fiber. The resulting synaptic outputs for each AN fiber type were summed to obtain the population response of this fiber type at each CF, which is comparable to the peri-stimulus time histogram (PSTH) used to describe experimental data. In order to analyze the steady-state encoding of the modulation, a 1-s long steady-state response, excluding on- and offsets, was analyzed. A fast Fourier transform (FFT) was performed on the resulting synaptic output and the magnitude value at the modulation frequency bin was considered the simulated EFR.

**Fig. 1:**
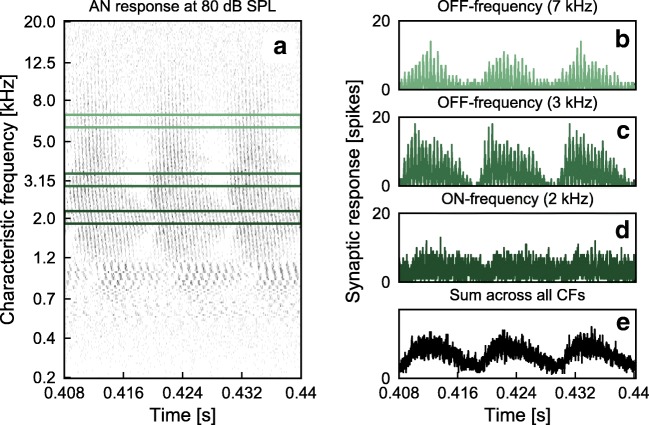
Simulated synaptic output of the AN model obtained using a SAM tone at 80 dB SPL with *f*_*c*_ = 2 kHz, *f*_*m*_ = 93 Hz and *m* = 0.85. A) Simulated AN synaptic output at CFs from 0.2 to 20 kHz for three cycles of the *f*_*m*_ at the steady-state part of the response. The green rectangles illustrate the on-frequency (2 kHz) and the off-frequency bands (3 and 7 kHz). B–D) Synaptic outputs at three different 1/3-octave bands. B–C) Off-frequency response at the bands centered at 7 and 3 kHz respectively. D) On-frequency response at the 2 kHz band. E) Synaptic output after summing across CFs

The model’s synaptic response was analyzed in populations corresponding to 1/3-octave frequency bands (CF bands) to investigate the contribution of each population to the total simulated AN EFR. The on-frequency (at or near the CF of the stimulus) simulated synaptic response was computed by summing the PSTH responses of all the CFs within the frequency band centered at 2 kHz. Similarly, contributions from the off-frequency bands centered at 3 and 7 kHz were calculated. Figure 1 shows an example of the simulated synaptic output. Panel A shows the response of the simulated AN at three cycles of the modulation frequency representing the sum of the three AN fiber types (high-, medium-, and low-SR) using a deeply modulated SAM tone at 80-dB SPL stimulus level (see the video animation for levels from 5- to 100-dB SPL in steps of 5 dB in the online-only version). Panels B–E show the simulated synaptic output at the output of the 1/3-octave band centered at 2 kHz (on-frequency, D), at the output of the 3-kHz (C) and the 7-kHz band (B), as well as summed across the entire frequency range (E). The summed synaptic output (E) was used to compute the simulated EFR to be compared to the recorded EFR.

Previous studies have attempted to simulate steady-state responses, such as EFRs (Rønne et al. 2013) or frequency following responses (FFRs) (Dau 2003), by convolving the simulated response of an AN model with a unitary response (e.g., Melcher and Kiang 1996) that reflected the contributions of different neural population along the auditory brainstem to the far-field evoked potential. In the present study, as CS occurs at the level of the AN, and for simplicity, only AN activity was considered (Zilany et al. 2009; Zilany et al. 2014). It was then assumed that the envelope encoding at the level of the AN would be similar to the recorded EFRs. It has been suggested though that EFRs to 80–100 Hz modulations are mainly generated at the level of the brainstem and midbrain (Herdman et al., 2002). However, Parthasarathy et al. (2016) showed good consistency between EFR recordings in rats and simulated EFRs using the cat version of the AN model of Zilany et al. (2009); Zilany et al. (2014).

## Results

The data reported in this study is publicly available online (Encina-Llamas et al. 2017b).

### EFR Magnitude-Level Functions in Human Listeners

**Fig. 2:**
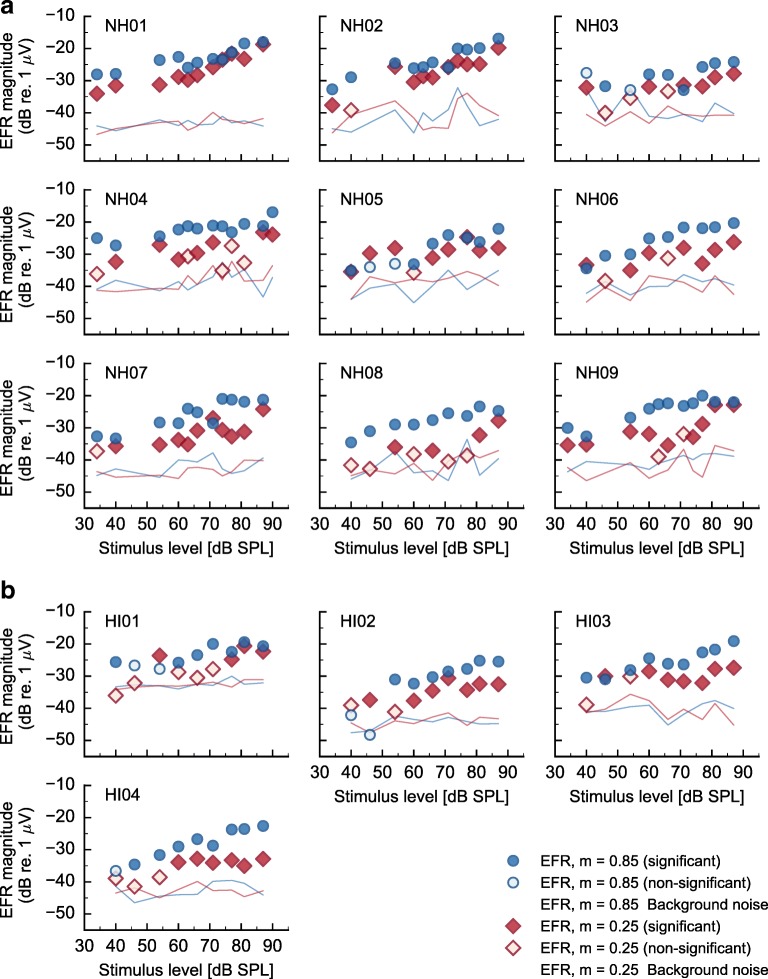
EFR magnitude-level function recorded in **a** NH threshold and **b** HI listeners using deeply (blue circles) and shallowly (red diamonds) modulated tones. EFR magnitudes in dB relative to 1 μV are represented as filled symbols in case of a statistically significant response (positive *F* test), and as open symbols in case of statistically non-significant (negative *F* test) responses. EEG background noises estimates for each modulation depth are shown as thin lines with consistent color labeling

Figure 2 shows the complete set of EFR magnitude-level functions for the NH threshold (A) and the HI (B) listeners. The recorded EFR magnitudes, represented in dB relative to 1 μV, are shown as blue circles for *m* = 0.85 or red diamonds for *m* = 0.25. Filled symbols represent statistically significant EFRs magnitudes (positive *F* test) and open symbols represent non-significant (negative *F* test) responses. The estimated EEG background noises for each modulation depth are depicted as thin lines with consistent color labeling.

For both listener groups, EFR magnitudes obtained with the deeply modulated stimuli (blue) were larger than those obtained with the shallowly modulated tones (red). However, different trends were observed in the EFR magnitude-level functions across listeners, particularly for the shallowly modulated tones. In the case of NH threshold listeners (Fig. 2a), the results are organized gradually from patterns showing monotonic and parallel EFR magnitude-level functions (i.e., listeners NH01 or NH02) to patterns showing non-monotonic magnitude-level functions (i.e., listeners from NH07 to NH09). In particular, for listener NH09, the EFR magnitudes for the deeply modulated tones grew monotonically with a constant linear slope throughout the whole level range. In contrast, the responses to the shallowly modulated tones initially grew with a single slope up to 55-dB SPL showed a decrease of the EFR magnitudes from 55- to 70-dB SPL, and then a recovery above 70-dB SPL, with comparable EFR magnitudes between 80- and 90-dB SPL as for the deeply modulated tones. Listener NH09 was considered as a potentially synaptopathic listener within the NH threshold group.

**Fig. 3:**
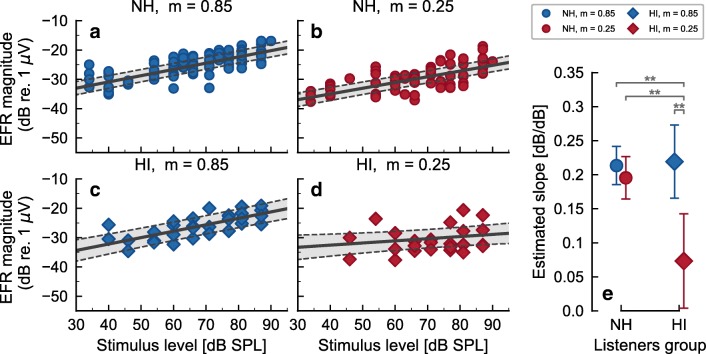
Estimated-marginal means fitted to the EFR magnitude-level functions recorded in the NH threshold (circles) and HI (diamonds) listeners using deeply (blue) and shallowly (red) modulated tones. Panels **a**–**d** show the EFR magnitude-level function for NH and deep modulation (**a**), NH and shallow modulations (**b**), HI and deep modulation (**c**), and HI and shallow modulation (**d**). Black solid lines in panels **a**–**d** represent the mean estimated slope for each listener’s group and modulation condition. Gray-shaded areas and black dashed lines represent the 95 % CI of the estimated mean. Panel **e** shows the estimated mean (with consistent symbols and color labeling) and 95 % CI for ease of comparison. Asterisks indicate statistical significance (** corresponds to a *P* ≤ 0.01)

For HI listeners (Fig. 2b), EFR magnitude-level functions for the deeply modulated tones grew monotonically with a similar slope as the NH threshold listeners. EFR magnitude-level function for the shallowly modulated tones showed, however, a strongly compressive or saturating growth. Figure 3 shows the fit of the statistical linear mixed-effects model to the EFR data for each listener group and modulation condition (panels a–d), and its corresponding mean estimated slopes predicted by the statistical model (panel e). Blue circles (a), red circles (b), blue diamonds (c), and red diamonds (d) represent the EFR magnitudes for NH and deep modulation, NH and shallow modulation, HI and deep modulation, and HI and shallow modulation respectively. The black solid lines in panels a–d represent the estimated mean slope from the statistical model, and the gray-shaded area and the black dashed lines represent the 95 % confidence intervals (CI). The estimated mean slopes and their 95 % CIs are shown again in panel e to allow for easy comparison.

A post hoc statistical analysis using multiple pairwise contrasts comparison of the estimated-marginal means revealed that the estimated slope in HI listeners for shallow modulations (d) were smaller (shallower) than the ones for HI listeners for deep modulation (c) [*t*_(187.17)_ = − 3.284, *P* = 0.0066], than the estimated slopes for NH listeners for shallow modulations (b) [*t*_(187.09)_ = − 3.171, *P* = 0.0095], and than the estimated slopes for NH listeners for deep modulations (a) [*t*_(187.06)_ = − 3.698, *P* = 0.0016]. The remaining comparisons were not found to be statistically different (*P* > 0.05).

### Simulating EFR Magnitude-Level Functions in Human Listeners With and Without Hair-Cell Dysfunction

**Fig. 4:**
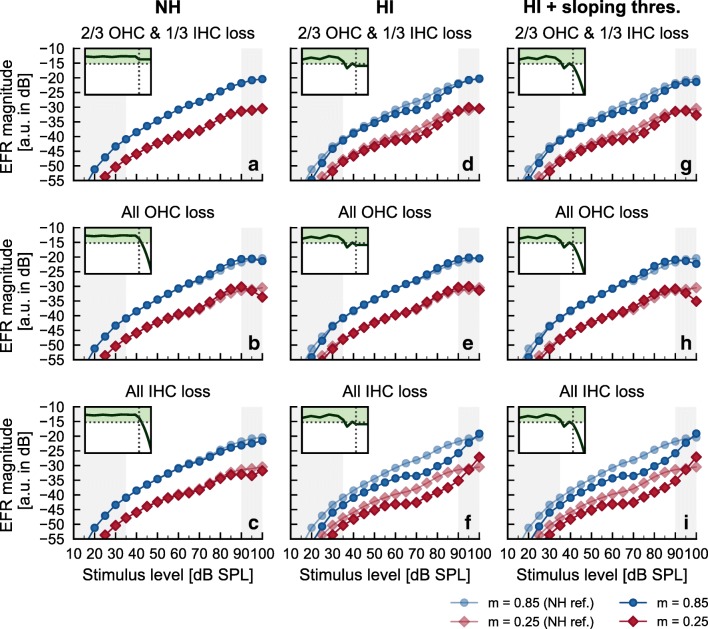
Simulated AN EFR magnitude-level functions in NH and HI listeners assuming only hair-cell dysfunction. First column (**a**–**c**) shows simulations for the NH listeners. Second and third columns (**d**–**i**) show simulations for the HI listeners. First row (**a**, **d**, and **g**) shows simulations assuming 2/3 of OHC and 1/3 of IHC dysfunction with flat EHF thresholds (**a** and **d**) and sloping EHF thresholds (**g**). Second row (**b**, **e**, and **h**) shows simulations assuming only OHC dysfunction with sloping EHF thresholds (**b** and **h**) and flat EHF thresholds (**e**). Third row (**c**, **f**, and **i**) shows simulations assuming only IHC dysfunction with sloping EHF thresholds (**c** and **i**) and flat EHF threshold (**f**). The insets in each panel show the simulated hearing thresholds as a function of frequency. The vertical black dotted lines in the insets indicate the 8-kHz frequency and the horizontal black dotted line and the green-shaded area indicate normal hearing thresholds (< 20-dB HL). Each panel provides a similar representation as in Fig. 2 but with the simulated EFR magnitudes expressed in arbitrary units (a.u.) in decibels. Simulated EFR magnitude-level functions are normalized to an average recorded EFR data point for easier comparison (see the text for details). Blue circles and red diamonds indicate simulated EFRs for deeply and shallowly modulated tones respectively. Semi-transparent lines and symbols in panels **b** to **i** are a reprint of the NH simulation shown in panel **a** for comparison. Gray-shaded areas indicate stimulus level ranges outside the recorded level range

Figure 4 shows the simulated EFR magnitude-level functions for NH and HI listeners assuming different degrees of OHC and IHC dysfunction (see Table 2 for the mean and standard deviation of the measured audiograms up to 8 kHz for each listener group). The representation is similar to Fig. 2, but with the simulated EFR magnitudes expressed in arbitrary units (a.u.) in decibels. Two postulated EHF audiometric threshold profiles (i.e., hearing thresholds beyond 8 kHz) were considered: constant thresholds beyond 8 kHz or a sloping function with a slope of about 50 dB/octave (Rodríguez Valiente et al. 2014). The insets in each panel of Fig. 4 show the hearing threshold assumed in each simulation. The vertical black dotted line in the insets represents the frequency at 8 kHz and the horizontal black dotted line and the green shaded area represent normal hearing threshold (< 20-dB HL). The semi-transparent lines and symbols in panels b–i represent the simulated EFRs for a NH listener assuming 2/3 of OHC and 1/3 of IHC loss (reprint of panel a for comparison).

**Table 2:** Mean (and standard deviations) of the audiogram values (in dB HL) for the NH threshold and HI listener groups. Audiogram values for the individual representative listeners NH01, NH09, and HI04

Frequency (kHz)	0.125	0.25	0.5	0.75	1	1.5	2	3	4	6	8
Mean NH group	0.6 (5.2)	2.2 (4.6)	0.6 (8.5)	3.3 (14.4)	2.2 (7.4)	− 1.0 (7.4)	0.0 (6.0)	0.7 (5.8)	0.6 (5.0)	0.6 (8.2)	8.3 (10.3)
Mean HI group	8.8 (7.5)	3.8 (4.8)	7.5 (6.3)	6.3 (6.3)	0.0 (4.1)	1.3 (6.3)	3.0 (6.3)	13.8 (7.5)	31.3 (2.5)	18.8 (16.5)	25.0 (17.8)
NH01	− 5	0	− 10		− 5	0	− 5		− 5	− 5	− 5
NH09	− 5	5	5	10	10	5	0	0	0	5	10
HI04	5	5	10	5	0	− 5	− 5	10	30	− 5	5

The first column (panels a–c) shows simulations for the NH group. The second and third columns (panels d–i) show simulations for the HI group. The first row (panels a, d, and g) shows simulations assuming a hair-cell loss distribution of 2/3 of OHCs and 1/3 of IHCs (e.g., Lopez-Poveda and Johannesen 2012; Spongr et al. 1997). The second row (panels b, e, and h) shows simulation results assuming only OHC dysfunction, and the third row (panels c, f, and i) shows simulation results assuming only IHC dysfunction. To facilitate the comparison with the recorded EFR data, the data point for the NH simulation at 80-dB SPL for *m* = 0.85 (panel a in Fig. 4) was fitted to the mean recorded EFR magnitude for the NH threshold listeners at 81-dB SPL for *m* = 0.85 (panel a in Fig. 3). The same normalization was applied to all simulated EFRs throughout this study.

The simulated EFR magnitude-level functions for NH listeners assuming a constant hearing threshold beyond 8 kHz and 2/3 of OHC and 1/3 of IHC dysfunction (panel a) showed a parallel and monotonic growth over the input level range used in the EFR recordings (35–90-dB SPL, unshaded area). The EFR magnitudes for the deeply modulated tones were larger than for the shallowly modulated tones. In general, the model simulations were able to capture the trend observed in the recorded EFR magnitude-level functions in some of the NH threshold listeners (i.e., NH01 and NH02, see Fig. 2a). Focusing on the effect of sloping EHF thresholds, neither a loss of only OHCs (panel b) nor a loss of only IHCs (panel c) has a notable effect on the EFR magnitudes over the level range used in the experiment. Elevated EHF thresholds result in a slight reduction of simulated EFR magnitudes at input levels above 90-dB SPL. Hence, the reduction of EFR magnitudes at mid input levels observed in some NH threshold listeners (e.g., NH09 in Fig. 2a) cannot be attributed to postulated elevated hearing thresholds at EHFs. It is worth commenting here that, any simulated hair cell damage leading to reduced EFR magnitudes, does it in a similar degree agnostic to modulation depth, in contrast to the recorded data. This is further discussed in the “On the quality and limitations of the AN model” section.

The simulated EFR magnitude-level functions for HI listeners (second and third columns in Fig. 4) show that (1) assuming only OHC dysfunction does not result in a change with respect to the NH group (panels a and b), regardless of the recorded mild threshold elevation at 4 kHz (panel e) or the additional postulated EHF threshold elevation (panel h). (2) Assuming only IHC dysfunction to account for the hearing threshold elevation at standard audiometric frequencies (below 8 kHz) results in a relatively small reduction of the simulated EFR magnitudes at mid-to-high input levels (panel f). And (3) additional postulated steep EHF threshold elevation does not result in further reduction of the simulated EFR magnitudes in any combination of hair-cell loss (second versus third columns). No combination of hair-cell dysfunction led to the saturated EFR magnitude-level functions for shallowly modulated tones observed in the experimental data (Fig. 2b). Hence, according to the AN model, such saturation cannot be attributed either to the measured elevated hearing thresholds at 4 kHz and beyond or to the postulated ones at EHFs.

### Simulating EFRs in NH Threshold Listeners and HI Listeners with Postulated CS

**Fig. 5:**
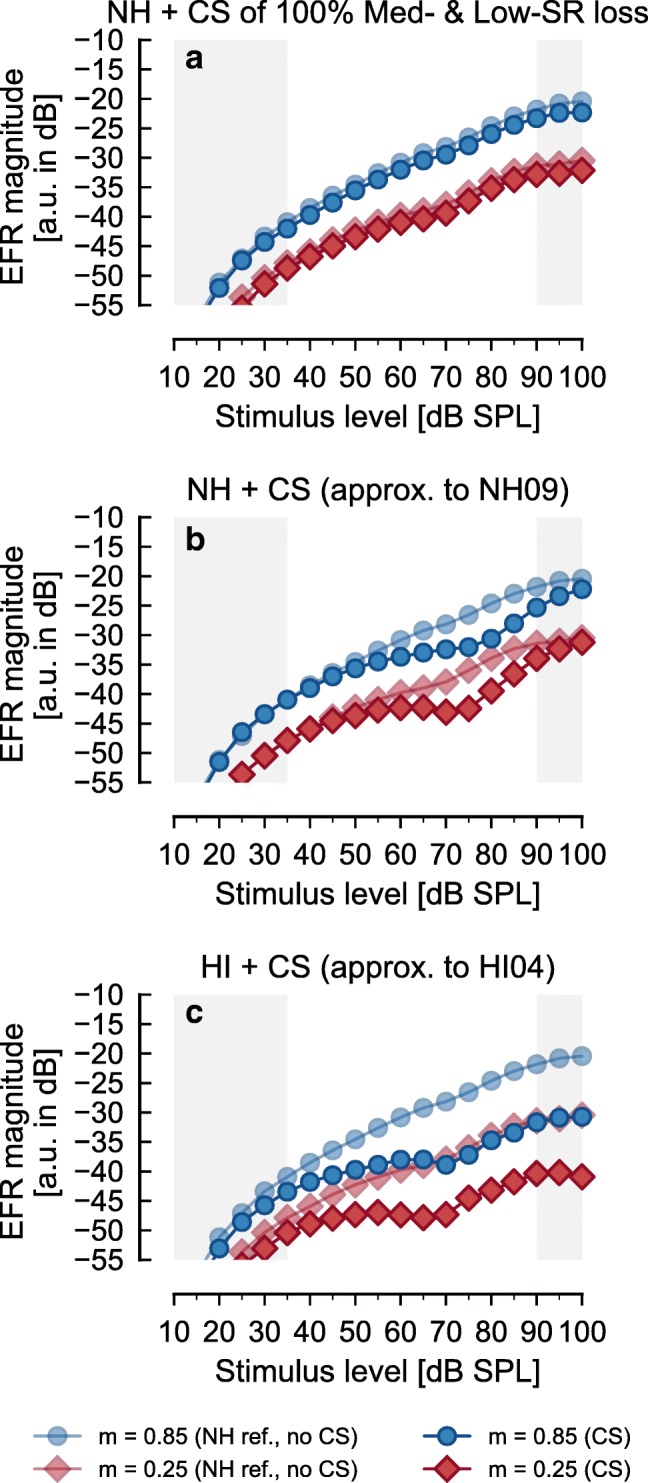
Simulated AN EFR magnitude-level functions in NH threshold and HI listeners when CS is included. Same representation as in Fig. 4. **a** Simulation for NH threshold with a loss of 100% of medium- and low-SR fibers only. **b** Simulation with a frequency-specific loss of all types of fiber synapses to approximate the response obtained in NH09 in Fig. 2a. **c** Simulation with a frequency-specific loss of all types of fiber synapses to approximate the response obtained in HI04 in Fig. 2b. Opaque lines and symbols show the simulated EFRs with additional CS, whereas the semi-transparent lines and symbols show the simulated EFRs for NH threshold listeners as reference (same as Fig. 4a)

Figure 5 shows the simulated EFRs for panel a NH threshold listeners after assuming a complete loss of medium- and low-SR fibers at all CFs; for panel b, a NH threshold listener including an empirically chosen synaptic loss that approximated the results obtained from listener NH09; and for panel c, a HI listener including an empirically chosen loss of synapses that approximated the results obtained from HI listener HI04. The representation is the same as in Fig. 4. The mean audiograms for the NH threshold listener’s group were used to set the modeled IHC and OHC parameters in the simulations shown in panel a. The audiograms of NH09 and HI04 were used to set the IHC and OHC parameters in the model shown in panels b and c. A combination of 2/3 of OHC loss and 1/3 of IHC loss and a flat EHF thresholds were assumed in all simulation results shown in Fig. 5.

The simulated EFR magnitude-level functions with a loss of 100 % of medium- and low-SR fibers (panel a, opaque lines and symbols) were nearly the same as the EFR magnitude-level functions in the reference simulation (semi-transparent lines and symbols), with a small decrement of less than 1.5 dB for both modulation depths. The non-monotonic growth found for some NH threshold listeners (i.e., NH09) required a frequency-specific loss of all types of AN fibers (panel b) and, more specifically, a loss of up to 85 % in the octave band centered at 4 kHz (range from about 2900 to 5600 Hz). In order to simulate EFR magnitude-level functions that are similar to those of the listener HI04 (panel c), a substantial loss of 85 % of all three types of AN fiber synapses at CFs above 2.5 kHz was required to be included in addition to the hair-cell dysfunction (with 2/3 of OHC loss and 1/3 of IHC loss). Simulated CS produced a reduction of the simulated EFRs compared to the NH threshold simulation (semi-transparent lines and symbols). For the recorded EFRs, a stronger reduction is observed for the shallowly modulated tones compared to the deeply modulated tones (see Fig. 2). In contrast, the impact of CS (see Fig. 5) and hair-cell dysfunction (see Fig. 4) on the model results was very similar for the deeply and shallowly modulated tones.

## Discussion

### EFR Magnitude-Level Functions from Deeply and Shallowly Modulated SAM Tones

It was hypothesized, via an heuristic argument, that CS produces differences in the EFR magnitude-level functions within a homogeneous group of young NH threshold listeners. It was further hypothesized that CS leads to non-monotonic or saturating EFR magnitude-level functions for the shallowly modulated SAM tones. This hypothesis was based on the assumption that high intensity sounds are dominantly encoded by the activity of medium- and low-SR AN fibers because the spiking rate of high-SR saturate at high stimulation levels (Liberman 1978; Yates 1990), as was previously proposed by Bharadwaj et al. (2014); Bharadwaj et al. (2015). Indeed, the individual results of NH threshold listeners demonstrated different EFR magnitude-level functions for shallowly modulated tones (Fig. 2a), and more similar functions for deeply modulated tones. For instance, the EFR magnitude-level function for the shallowly modulated tones grew monotonically with a single slope for the listener NH01, whereas for NH09, the EFRs grew non-monotonically, with reduced magnitudes at medium stimulus levels but larger magnitudes at higher levels. The EFR magnitude reduction at 65–70-dB SPL was about 10 dB. This difference might reflect a physiological difference between those two subjects because it is larger than the estimated intrinsic EFR variability. Specifically, in a previous study (Encina-Llamas et al. unpublished; Encina-Llamas et al. 2017a), we found that the test-retest variability in EFRs at 70-dB SPL from SAM tones at *f*_*c*_ = 2000 Hz, *f*_*m*_ = 93 Hz and *m* = 0.85 was 5 dB or less, much smaller than the difference seen across NH threshold listeners in Fig. 2. There are no studies to our knowledge specifically addressing whether test-retest reliability might alter with modulation depth. For the discussion here we are going to assume that this is not the case. Whether the different patterns observed in the EFR magnitude-level functions in listener NH01 versus NH09 are due to a loss of AN fiber synapses is unknown. It should be emphasized that, although the average hearing threshold of NH09 was about 8.5-dB HL larger than that of NH01 (see Table 2), both listeners are within the NH threshold limits (i.e., ≤ 20-dB HL).

Individual differences in the EFR magnitude-level functions for shallowly modulated tones were also observed for HI listeners (Fig. 2b). Whereas the EFR magnitude-level functions for deeply modulated tones grew monotonically with a single slope (similarly to the deeply modulated EFRs in NH threshold listeners), the EFR magnitudes for shallowly modulated tones did not vary much across stimulus level, leading to a saturated growth function. The change in slope was shown to be statistically significant (linear mixed-effects model, Fig. 3). Listener HI04 was considered representative within the HI group. It should be noted here that the main difference between NH09 and HI04 was a mild threshold elevation of 30-dB HL at the 4-kHz audiometric frequency in HI04. At other measured audiometric frequencies, both listeners showed thresholds within the NH range, and the average hearing threshold of HI04 was less than 1-dB HL larger than the one from NH09. However, the EFR magnitude-level functions from shallowly modulated tones for NH09 and HI04 were quite different. In fact, listeners NH01, NH09, and HI04 had hearing threshold within the NH threshold range at all audiometric frequencies except at 4 kHz; and yet, although their EFR magnitude-level functions for deeply modulated tones are comparable, the EFRs for shallowly modulated tones exhibit markedly different trends.

### A Model of the Auditory Nerve to Investigate Individual Differences in EFR Magnitude-Level Functions

**Fig. 6:**
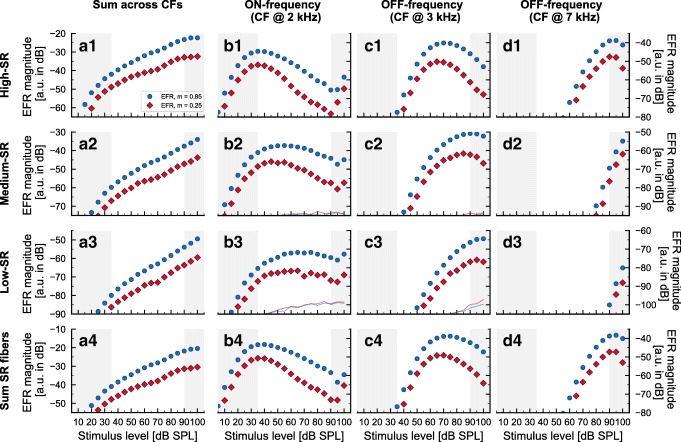
Simulated AN EFR magnitude-level functions in a NH listener, separately for different CF bands and for each AN fiber type. Solid circles represent statistically significant EFR magnitudes in decibel and open circles represent non-significant responses. Blue markers show responses for deeply modulated stimuli and red markers for shallowly modulated stimuli. The thin lines represent background noise (which falls below the smallest plotted values for many panels). The columns show the EFR magnitude-level functions centered at different CF bands and the rows show the simulated results for the different AN SR fiber types. Note the different dynamic ranges on the ordinate

Under the assumption that the EFR can be predicted from the summation of the instantaneous firing rate of AN fibers across frequency and fiber type, model simulations should help to shed light on the contributions of CF bands and fiber type to the overall response. Figure 6 shows the simulated EFR magnitude-level functions for a NH listener, separately for each fiber type (rows) and CF band (columns). Column a (panels a1–a4) shows the synaptic output summed across all CF bands. Column b (panels b1–b4) shows the output of the band centered at 2 kHz (on-frequency band). Column c (panels c1–c4) shows the output of the band centered at 3 kHz, and column d (panels d1–d4) the output of the band centered at 7 kHz.

The simulated AN activity to SAM tones in quiet at different CF bands (columns) showed that the simulated EFRs at medium-to-high stimulus levels were not purely due to activity in the on-frequency band (column b), but had strong contributions from AN neural populations located more basally (i.e., at higher CF bands, columns c and d). Within each frequency band, the response showed a bell-shaped curve, horizontally shifted along the stimulus level axis for more distal CF bands. This is consistent with the synchrony-level functions recorded from single neurons in the AN of the cat reported in Joris and Yin (1992). Hence, at higher stimulation levels, the off-frequency contributions dominated the total EFR magnitudes to SAM tones in quiet in the model framework, leading to the overall monotonic growth observed when summing across CFs.

Similarly, when analyzing the contributions of the different types of AN fibers (rows in Fig. 6), the simulated EFRs were dominated by the high-SR fibers across the whole stimulus level range. Focusing on the on-frequency band (column b) for simplicity, the medium-SR (b2) and low-SR (b3) fibers level-growth functions increased more rapidly with level than did the high-SR (b1) fibers; however, their overall response to the modulation frequency of the SAM tone was still smaller in an absolute sense than those of the high-SR fibers (notice the different ordinate axis scales). As a result, the medium- and low-SR fibers contribute very little to the total summed response (b4). Because the distribution of the three AN fiber types is unknown in the human, the fiber distribution from the cat (Liberman 1978) was implemented in the AN model. It should be noted, though, that the uneven distribution of the different fiber types (about 60 % of high-SR versus about 40 % of medium- and low-SR fibers) cannot fully explain the dominance of the high-SR fibers in simulated responses. A distribution ratio of 1.5 cannot account for differences in the simulated EFR magnitudes between the high-SR fibers (a1) and the sum of medium- and low-SR fibers (sum of panels a2 and a3) of 10–20 dB. An additional simulation (not shown) with only one fiber of each type per CF also showed a similar dominance of the high-SR fibers over the other two AN fiber types. The high-SR fiber dominance in the simulated AN EFR likely comes in part from their higher spiking rate. The spiking rate of the sum of medium- and low-SR fibers is only comparable to that of the high-SR fibers in the on-frequency band at high supra-threshold input levels. The rate of medium- and low-SR fibers at low-to-medium input levels in the on-frequency band and across the whole input level range at off-frequencies is much lower than the rate of high-SR fibers. There is, however, some evidence that low-SR fibers may have more synaptic connections to higher stages of the auditory pathway than high-SR fibers (Liberman 1991; Rouiller et al. 1986). This greater number of synaptic connections might increase the importance of low-SR fibers in auditory processing. If this finding also applies to humans, this has to be considered when attempting to relate electrophysiological gross-potentials to psychoacoustical performance. On the other hand, even if this was true, the functional relevance of low-SR fibers in encoding fluctuations has been questioned based on its spiking rate statistics (Carney 2018).

In conclusion, the AN model simulations suggest that, as the envelope of SAM tones in quiet is better encoded at on-CFs at low input intensities and at off-CFs at higher input levels (see also Fig. 1), the high-SR fibers always dominate the overall EFR for this stimulus condition.

### Effect of Hair-Cell Dysfunction

The simulations provide an explanation for the insensitivity of the simulated EFR magnitude-level functions to a pure OHC dysfunction (Fig. 4, second row). HI listeners only had audiometric thresholds above 20-dB HL at frequencies beyond 3 kHz. Sloping threshold elevation was postulated for EHFs beyond 8 kHz. A loss of gain (OHC dysfunction) underlying the increased thresholds would only affect the response when the stimulus frequency is close to the CF of the corresponding fiber (on-frequency processing) because OHC dysfunction results mainly in a reduction of sensitivity at the tip of the tuning curve (leading to broader frequency selectivity). However, the tails (off-frequency) will be largely unaffected, apart from some modest hypersensitivity (Liberman and Dodds 1984). Thus, the responses of AN fibers with high CF tuning excited by stimuli with energy at lower CFs (off-frequency excitation) remain essentially independent of OHC loss. This explains the result of the simulated EFRs assuming only OHC dysfunction (panels b, e, and h in Fig. 4). In contrast, IHC dysfunction results in a general loss of sensitivity (both at the tip and at the tail) resulting in an elevated AN tuning curve (Liberman and Dodds 1984). In the model, the largest effect was found when only IHC dysfunction was considered (Fig. 4f); there was an intermediate effect if hair-cell dysfunction was assumed to affect 2/3 of OHCs and 1/3 of IHCs (Fig. 4d). Consistent with the discussion in the “A Model of the Auditory Nerve to Investigate Individual Differences in EFR Magnitude-Level Functions” section, a threshold elevation due to IHC dysfunction at a frequency range between 3 and 8 kHz will result in a perturbation of the envelope coding of SAM tones with carrier frequency at 2 kHz for input levels of about 60 to 90-dB SPL, similar to the effect observed with CS at this frequency range.

The postulated sloping hearing threshold elevation beyond 8 kHz had a minimal impact (in comparison to the postulated flat EHF threshold) on the overall EFR magnitude-level functions (panel a vs b and c, and second vs third column in Fig. 4). The sloping threshold elevation only produced a small reduction at input levels above 90-dB SPL (outside the recorded level range).

It should be noted that in all the cases where simulated hair-cell dysfunction resulted in reduced EFR magnitudes, the reduction was observed for both deeply and shallowly modulated tones. This is at odds with the recorded EFR magnitude-level functions in some NH listeners and in the HI listeners, where a reduction of EFR magnitudes was only observed for shallowly modulated responses. This is a general observation in the entire modeling approach, including in simulations with additional postulated CS, as discussed below.

In summary, no combination of IHC and/or OHC hair-cell impairment led to simulated EFR magnitude-level functions similar to the recorded EFR data, indicating the need for including additional damage in the model.

### Effect of Additional Postulated Synaptic Loss

The model framework was used to explore whether additional synaptic loss explained the non-monotonic patterns observed in some NH threshold listeners (Fig. 2a) and the strong saturation found for HI listeners (Fig. 2b). Even though part of the analysis is performed on individual representative listeners, we are not trying to claim that a given listener suffers from CS. Our purpose was to investigate the potential effects of postulated CS on the EFR magnitude-level functions using the modeling framework.

The reanalysis of the data from Furman et al. (2013) by Marmel et al. (2015) suggested that synaptic loss affected medium- and low-SR fibers more than high-SR fibers, but not that high-SR fibers were unaffected. This is consistent with the simulation results showing that a synaptic loss of high-SR fibers is required. The model simulations presented in panel a of Fig. 5 show that the simulated EFR magnitude-level functions are unchanged if only medium- and low-SR fibers are lost. A reduction of less than 1.5 dB was obtained in the simulated EFRs after including a complete loss of the medium- and low-SR fibers at all CFs. Due to the variability of the recording method, such a small reduction would not be detectable in EFR recordings within a realistic time frame. However, in non-human animal studies in which CS was demonstrated and quantified through histological analysis, but in which the type of AN synapse loss was not clearly identified, EFRs were shown to be reduced in noise-exposed synaptopathic mice relative to unexposed animals (Shaheen et al. 2015) and in older animals (with more CS) relative to younger ones (Parthasarathy and Kujawa 2018).

**Fig. 7:**
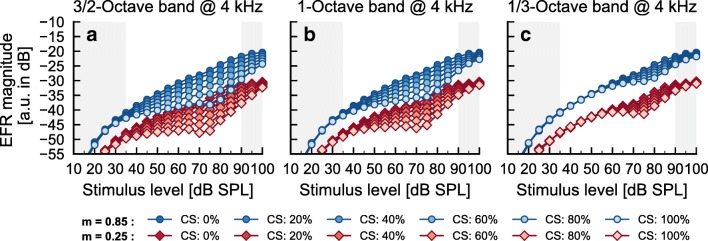
Analysis of the effect of BW and degree of CS on the simulated AN EFR magnitude-level functions. Same representation as in Figs. 4 and 5). **a** Simulation with CS at a BW of 3/2-octave band centered at 4 kHz. **b** Simulation with CS at a BW of 1-octave band centered at 4 kHz. **c** Simulation with CS at a BW of 1/3-octave band centered at 4 kHz. Gradual coloring (blue for deeply modulated tones and red for shallowly modulated tones) in all panels represent the degree of CS ranging from 0 % (NH, as in Fig. 4a), 20 %, 40 %, 60 %, 85 %, and 100 %

In order to obtain significant reductions of simulated EFR magnitudes, some degree of high-SR fiber loss had to be included in the AN model framework (Fig. 5b, c). The non-monotonic growth found in some NH threshold listeners (i.e., NH09) and the shallow growth or saturation found in HI listeners (i.e., HI04) could then be accounted for by reducing the number of all types of AN fibers (including high-SR fibers loss) at off-frequency CFs. A qualitative analysis of the simulated EFR magnitude-level functions at the on- and off-frequency bands (see Fig. 6) showed that the off-CF fibers dominated the total (summed across-CF) simulated EFRs in the level interval at which they were reduced in the synaptopathic NH simulation (Fig. 5b; opaque lines and symbols) compared to the NH simulation without CS (Fig. 5b; semi-transparent lines and symbols). The roles of CF bandwidth (BW) and degree of CS on the simulated responses in explaining the data from NH09 were investigated. Figure 7 shows simulated EFR magnitude-level functions with BWs including CS of 3/2-octave (panel a), 1-octave (panel b), and 1/3-octave (panel c) centered at 4 kHz (based on the qualitative analysis of the simulated EFR magnitude-level functions at off-CFs) for deeply (blue circles) and shallowly (red diamonds) modulated tones. The color gradient indicates different degrees (percentage) of synaptic loss from 0 % (NH) to a loss of 100 % of AN fibers. The results show that the range of input levels over which the simulated EFRs were reduced was larger as the BW of CS increased, and that the reduction of the simulated EFR magnitudes increases with the percentage of synaptic loss.

The experimental EFR data showed some variability that could be explained by the BW of CS. The EFR magnitudes for shallowly modulated SAM tones presented at around 75-dB SPL for NH09 were about 8–10 dB lower than for NH01 (Fig. 2a). The EFR magnitudes for NH09 diverged from those of NH01 between 55–60- and 80–85-dB SPL but converged again at input levels above 80–85-dB SPL. A BW of 1-octave and a loss of 85 % were considered to be suited for NH09 because (1) the range of levels at which the simulated EFR magnitudes were reduced fell between 55- and 85-dB SPL, (2) the reduction of the simulated EFR magnitudes was maximal assuming a limit of 85 % synaptic loss (see below), and (3) the simulated EFR magnitudes at levels above 85-dB SPL and below 55-dB SPL did not diverge significantly from the simulation without CS.

The similarity of the model outputs and the measured data could be slightly improved and made more physiologically plausible by assuming a smoother transition between the non-synaptopathic frequency ranges and the synaptopathic one (see Table 3). The considered solution was limited by two constraints in order to accommodate physiological findings. First, a limit of 85 % of synaptic loss was set based on a non-human animal study that reported that a loss of 85 % of IHCs produced hearing threshold shifts of less than 20 dB (Lobarinas et al. 2013). Second, a maximum total (across CF) AN synapse loss of < 20 % (the total synaptic loss for NH09 was 17.8 %) was set based on the age of the listener (30 years) and a recent study quantifying AN fibers survival with age in human cadavers (see Fig. 9 in Wu et al. 2018). This solution led to a reduction of the simulated EFR magnitude-level functions between 55 and 60 and 85-dB SPL, with a maximum reduction of about 6.5 dB at 75-dB SPL and almost identical simulated EFR magnitudes (with respect to the NH simulation reference as semi-transparent lines and symbols) for the shallowly modulated tones at input levels below 55-dB SPL and above 90-dB SPL (Fig. 5b). This was similar to the recorded data (Fig. 2a). In contrast to the recorded EFR magnitude-level functions, the reduction in the simulated EFR magnitudes was, however, observed for both modulation depths.

**Table 3:** Percentage of all three types of additional AN fiber loss at different CFs implemented in the model in NH threshold and HI listeners’ simulations

Frequency (kHz)	≤ 2	2.3	2.4	2.5	2.7	2.8	2.9	3	4	5.65	5.7	5.75	≥ 6
Loss of AN fibers (%) in best approx. to NH09	0	0	0	0	20	50	85	85	85	85	50	20	0
Loss of AN fibers (%) in best approx. to HI04	20	30	50	85	85	85	85	85	85	85	85	85	85

In order to approximate the results of the HI listeners, a large loss of all three types of AN fiber synapses had to be included in a broad off-CF range. The recorded EFR magnitudes for shallowly modulated tones in HI04 were almost constant from 60 to about 90-dB SPL input levels (Fig. 2b). The analysis in Fig. 7 showed that extending the loss of synapses towards higher CFs (with respect to the solution for NH09) leads to a further reduction of the simulated EFR at higher input levels (Fig. 5c). As HI04 was 67 years old, the limit of total synaptic loss was set to 60 % (the total synaptic loss for HI04 was 57.3 %), based on Wu et al. (2018). This solution led to simulated EFR magnitude-level functions with a very shallow growth at input levels from 40- to 90-dB SPL. As for the NH listeners, the modeled CS affected the simulated EFRs similarly for both deeply and shallowly modulated tones, which is at odds with the recorded data.

The simulations do not prove that listener NH09 and HI04 suffer from a loss of AN synapses like those found in the modeled solutions. In fact, although a loss of up to 85 % of IHCs in rats did not result in thresholds shifts larger than 20 dB (Lobarinas et al. 2013), it seems unlikely (although this is speculation) that a severe loss of 85 % of AN fibers in a considerable range of CFs would not lead to some sort of noticeable deficit in perception. As an observation during the experiment, none of the recorded listeners complained about having hearing difficulties in challenging acoustic scenarios and all of them were able to maintain a normal conversation in the quiet lab environment without showing noticeable difficulties.

### On the Quality and Limitations of the AN Model

The AN model by Zilany et al. (2009); Zilany et al. (2014) is able to account for a large range of reported physiological data from single-unit recordings in cats. Crucial to this study is the performance of the AN model for supra-threshold stimulation and responses to off-CF AN activity. Regarding supra-threshold stimulation, Zilany and Bruce (2006) (using an earlier version of the AN model used here, in the present study) showed that the AN model is able to faithfully capture AN tuning curves (e.g., Liberman 1978), the sharpness of tuning represented by *Q*_10_ values at different CFs, and the best threshold curve (Miller et al. 1997). Physiological recordings from the cat showed that OHC dysfunction leads to elevated thresholds at the tip of the tuning curves and hypersensitivity in their low-frequency tails (e.g., Liberman 1984; Liberman and Dodds 1984; Liberman and Klang 1984). The model can correctly account for these effects of OHC dysfunction (see Fig. 5 in Zilany and Bruce 2006). Modeled rate- and phase-level curves for the unimpaired condition and with impairment of OHCs and/or IHCs (see Fig. 6 in Zilany and Bruce 2006) are also consistent with physiological results in the cat (Heinz and Young 2004; Liberman and Klang 1984; Miller et al. 1999). Regarding the AN model response to modulation frequency encoding (or synchrony) at on- and off-frequencies, Zilany et al. (2009) showed a good consistency between the model responses to amplitude modulated tones (see the section Results E in Zilany et al. 2009) and physiological data recorded in AN fibers in the cat (Joris and Yin 1992). The model correctly captures the gain and synchrony to *f*_*m*_ as a functions of modulation depth, envelope synchrony as a function of input level (similar to the model responses shown in Fig. 6, b1), and modulation transfer functions (MTF) of high-CF fibers. However, the AN model has some limitations with respect to the MTF cutoff frequency and the AN fiber tuning-curve parameters (CF and bandwidth); specifically, the model shows a saturation at the MTF corner frequency at higher CFs (see Fig. 15 in Zilany et al. 2009). More relevant to this study, the AN model shows a higher maximum synchrony to *f*_*m*_ than the physiological data for high-SR AN fibers tuned to high-CFs (> 5 kHz). This may lead to an over-representation of the envelope encoding of high-SR fibers tuned to high-CFs, which may enhance the dominance of the off-frequency contributions in the present study. Finally, the version of the AN model by Zilany et al. (2014) was adjusted to correctly account for the AN discharge rate at saturation reported in Liberman (1978), improving the overall modeled AN response across CF compared to previous versions of the model (see Fig. 1 in Zilany et al. 2014).

Although the simulated EFR magnitude-level functions were generally consistent with the trends observed in the recorded EFRs, the model was not able to capture all of the details observed in the recorded data. The implementation of either IHC dysfunction or CS within the model framework similarly affected the predicted EFR magnitude-level functions for both deeply and shallowly modulated tones (see Figs. 4 and 5). In contrast, the recorded EFR level-growth functions for deeply modulated tones were similar for all listeners (both NH threshold and HI); substantial individual differences were observed only for the EFRs in response to shallowly modulated tones (see Fig. 2). Consistent with the AN model simulations, Shaheen et al. (2015) reported a significant reduction in the EFR amplitude-level functions of synaptopathic mice when using deeply modulated SAM tones with modulation frequencies between 800 to 1000 Hz. The EFRs in mice showed group delays consistent with generators between the AN and the cochlear nucleus. In the present study, a modulation frequency of 93 Hz was used to elicit the EFRs in humans, assumed to be mainly dominated by brainstem sources at this *f*_*m*_ (Herdman et al. 2002; Kuwada et al. 2002). Brainstem processing, such as central gain mechanisms (Chambers et al. 2016; Möhrle et al. 2016), that affect deeply and shallowly modulated stimuli differently, may explain the inconsistency of the human data versus the non-human animal data and the model simulations. A recent study in rats reported enhanced EFR amplitudes in response to SAM tones in quiet at a high supra-threshold level for deep modulation depths (*m* = 1) but not for shallow modulation depths (*m* = 0.25) in aged animals (Lai et al. 2017), consistent with an animal model of age-related CS (Parthasarathy and Kujawa 2018; Sergeyenko et al. 2013). A central gain compensatory mechanism that restores EFR magnitudes for deeply modulated tones but not for shallowly modulated tones could explain why EFR magnitudes at the level of the AN in the synaptopathic AN computer model (Fig. 5) and in mice (Shaheen et al. 2015) are reduced at both modulation depths, while the reduced EFRs were only observed for shallowly modulated responses in human listeners who may have CS (Fig. 2).

### Considerations on the Use of EFRs to Investigate CS in Future Studies

Model simulations suggested two main conclusions when SAM tones in quiet were used as sound stimuli: (1) the EFR magnitude-level functions at medium-to-high stimulation levels are strongly dominated by the contributions from off-frequency neuronal activity, and (2) there must be a significant degree of loss of high-SR fibers for CS to be reflected in the EFR.

First, the interpretation of the role of the medium- and low-SR fibers on encoding temporal fluctuations at high stimulus levels, based on the rate-level curves of the different AN fibers types (Liberman 1978; Yates 1990), has led to different hypotheses when studying CS in humans (including the present study and others like Bharadwaj et al. 2014; Bharadwaj et al. 2015; Marmel et al. 2015; Mehraei et al. 2016; Paul et al. 2017). AN rate-level functions are derived from direct recordings in single AN neurons, and therefore provide information regarding AN neuronal activity at on-frequency stimulation. However, electrophysiological evoked responses reflect the synchronized activity of large neural populations. As CS affects supra-threshold processing, high sound stimulation levels that produce a broad excitation of the AN are commonly used. Thus, the contributions of AN neurons tuned to off-frequency CFs should be carefully considered in the design of future hypotheses, in particular when narrow-band stimuli are presented in quiet. To take this into account, other authors have proposed the use of background masking noise to attenuate the effect of the off-frequency contributions. For example, notch-noise maskers at SNR of 20 dB and broadband maskers at SNR of 10 dB were used by Bharadwaj et al. (2015) and Mehraei et al. (2016), respectively. It is known that presenting broader and more complex stimuli with energetic content at off-frequency CFs in addition to a single SAM tone in quiet (e.g., SAM tones in masking noise or multiple SAM tones with different carrier frequencies) might attenuate off-frequency contributions compared to a single SAM tone. However, this likely depends on the relative spectral density of the SAM-generated off-frequency excitation and the excitation produced by the masking noise. At supra-threshold levels, the envelope synchrony of AN neurons is larger at off-frequency CFs than on-frequency, suggesting that high levels of masking noise may be needed to obtain a response restricted to on-frequency AN components. In-depth modeling and experimental analysis of the use of masking noise to maximally attenuate off-frequency contributions could be of interest when using EFRs to investigate CS.

Second, an over-simplified interpretation of the data in Furman et al. (2013) posits that only medium- and low-SR fibers are affected by CS, although a reanalysis of the data already concluded that in fact there was also a significant loss of high-SR fibers (Marmel et al. 2015). The results obtained with the AN model support the view that high-SR fibers are affected by CS. A significant loss of high-SR fibers is needed to obtain reduced simulated EFR magnitudes. The idea that CS also involves high-SR loss is consistent with the findings from Bourien et al. (2014), who showed that changes in ABR wave-I amplitudes, similar to the reduction in ABR wave-I amplitudes observed in synaptopathic non-human animals (e.g., Furman et al. 2013; Kujawa and Liberman 2009; Shaheen et al. 2015), are more likely to be due to loss of high-SR fibers rather than of medium- and low-SR fibers. In addition, our model simulations are consistent with previous modeling findings (Paul et al. 2017), where a certain degree of high-SR fiber loss had to be included to account for the differences observed in the EFR magnitudes recorded in NH threshold listeners with and without tinnitus, which has also been related to CS (Bramhall et al. 2018; Schaette and McAlpine 2011).

## Conclusions

EFR magnitude-level functions recorded from a group of young NH threshold listeners showed individual differences for deeply and shallowly modulated tones, indicating differences in neural supra-threshold encoding of envelope modulations. Similar differences for mild HI listeners measured at an audiometrically normal center frequency supported the idea of coexisting hearing loss due to hair-cell dysfunction and supra-threshold deficits at frequencies of normal sensitivity.

A model of AN activity was able to account for the monotonic growth with level observed in the recorded EFR magnitude-level functions of the NH threshold listeners. Hair-cell dysfunction, with or without a postulated steep sloping threshold elevation at extended audiometric frequencies beyond 8 kHz, was not sufficient to explain the non-monotonic trends obtained in the EFR data for some of the particular NH threshold listeners. Similarly, hair-cell dysfunction alone could not account for the EFR data recorded in the HI listeners. This suggests that additional damage, namely CS, must be included in the model to account for the recorded EFRs. A loss of all types of AN fibers (including high-SR fibers) at a specific cochlear frequency range needed to be implemented in the model to account for the data of some NH threshold listeners showing reduced EFR magnitudes at mid-stimulation levels. A loss of exclusively medium- and low-SR fibers had no impact on the simulated EFR magnitude-level functions, which were essentially the same as those obtained in non-synaptopathic simulations. The same was found for CS in HI listeners, where a large loss of all three AN fiber types had to be included in a broad CF range to match measured results.

Overall, the data and the simulations suggest that, when using SAM tones in quiet as sound stimuli, EFRs are dominated by high-SR fibers, and that off-frequency neurons increasingly contribute to the EFR with increasing stimulus level. The finding that the envelope is better encoded at off-frequency CFs (rather than on-frequency) when SAM tones in quiet are presented at high stimulus levels must be considered when using EFRs to investigate supra-threshold coding with these stimulus paradigms. An in-depth modeling and experimental analysis on the effect of noise makers to fully attenuate off-frequency contributions could be of interest when investigating the use of EFR to diagnose CS in living human listeners. In addition, parallel electrophysiological studies in humans and non-human animals where CS has been characterized (e.g., mice), together with the use of species-specific computational models, are needed to quantify the potential consequences of CS in humans.

## Electronic Supplementary Material

ESM 1 (MOV 4629 kb)
